# Inhibition of B16 melanoma growth and metastasis in C57BL mice by vaccination with a syngeneic endothelial cell line

**DOI:** 10.1186/1756-9966-28-13

**Published:** 2009-01-31

**Authors:** Kenta Yoshiura, Toshihide Nishishita, Takashi Nakaoka, Naohide Yamashita, Naomi Yamashita

**Affiliations:** 1 Research Institute of Pharmaceutical Sciences, Musashino University, Shinmachi 1-1-20, Nishitokyo-shi, Tokyo 202-8585, Japan; 2 Department of Advanced Medical Science, Institute of Medical Science University of Tokyo, Shirokanedai 4-6-1, Minato-ku, Tokyo 108-8639, Japan

## Abstract

**Background:**

Key role of angiogenesis in tumor growth and metastasis based on accumulating evidence and recent progress of immunotherapy have led us to investigate vaccine therapy targeting tumor angiogenesis.

**Methods:**

C57BL/6J mice were vaccinated with a syngeneic endothelial cell line Tpit/E by subcutaneous injection once a week. Prior to ninth vaccination, the mice were challenged with B16/F10 melanoma cells by subcutaneous inoculation on the back for the tumor growth model or by tail venous injection for the lung metastasis model. Development of subcutaneous tumor and lung metastasis was monitored by computed tomography scanning, which enabled accurate evaluation with the minimized sacrifice of mice.

**Results:**

Vaccination with Tpit/E cells inhibited subcutaneous tumor growth and appearance of lung metastasis compared to control. Survival period was elongated in the Tpit/E vaccination in both of the two models. We also obtained hybridomas secreting specific antibodies to Tpit/E cells from a mouse vaccinated with the cells, indicating that specific immune response to the syngeneic endothelial cells was elicited.

**Conclusion:**

These results suggest that vaccination with an autologous endothelial cell line may be effective against melanoma.

## Background

Cancer immunotherapy has now gained importance as therapeutics especially for cancers resistant to surgery, chemotherapy or radiation therapy. Previously, we have shown that melanoma patients vaccinated with tumor lysate pulsed-dendritic cells elicited antibody response to carbonic anhydrase II of which expression was specific to tumor endothelial cells [1]. Angiogenesis has been shown to play a key role in tumor growth and metastasis and new molecules targeting tumor angiogenesis have been discovered and coming into clinical use [2-5]. These findings have led us to investigate cancer vaccine therapy targeting tumor angiogenesis.

Efficacy of immunotherapy targeting known molecules associated in tumor angiogenesis such as VEGF [6], VEGFR-2 [7-10], FGF-2 [11], FGFR-1 [12], endoglin [13], Tie-2 [14], HP59 [15], survivin [16], matrix metalloproteinase [17], integrin beta3 [18], vascular endothelial-cadherin [19], angiomotin [20], and angiopoietin-2 [21] have been reported. Many other immunogenic antigens associated in tumor angiogenesis remains to be explored for the relevance as a target of immunotherapy.

Immunotherapy targeting tumor vasculature appears to have advantages over conventional immunotherapy targeting cancer cells, as it is assumed that failure of antigen-presentation mechanism, decrease of antigenicity by frequent mutation seen in cancer cells do not occur in vascular endothelial cells and that access of effectors is much easier in targeting vascular endothelium. So far, several reports have shown that tumor growth and metastasis were inhibited by vaccination with whole endothelial cells in mice [22-24]. Among these reports, a syngeneic sinusoidal endothelial cell vaccine has been shown to be effective in BALB/c mice [23]. In the present study, we tested an immortalized syngeneic endothelial cell line originated from C57BL mice as a vaccine for treatment of melanoma. Advantages in use of a cell line are as follows: proliferating cells in culture may share angiogenic antigens with tumor vascular endothelial cells [25], and a cell line is able to supply as many cells as necessary with ease. Here, we demonstrated that vaccination with a syngeneic endothelial cell line Tpit/E inhibited growth and metastasis of B16/F10 melanoma. We also obtained hybridomas secreting specific antibodies to Tpit/E cells from the vaccinated mouse to prove occurrence of the specific immune response to the syngeneic endothelial cells.

## Methods

### Cell lines and culture

B16/F10 melanoma and SP-2 myeloma cell lines were provided by Cell Resource Center for Biomedical Research Institute of Development, Aging and Cancer Tohoku University (Sendai, Japan), and cultured in RPMI-1640 (Invitrogen, Carlsbad, CA) with 10% fetal bovine serum (FBS; Thermo Trace Ltd, Melbourne, Australia), at 37°C in an atmosphere of 95% air and 5% CO_2_. Tpit/E vascular endothelial cell line derived from pituitary gland of temperature sensitive T-antigen transgenic mouse was provided by RIKEN BRC Cell Bank (Tsukuba, Japan), and cultured in HAMF12/DMEM (Invitrogen, Carlsbad, CA) with 10% horse serum (Nichirei, Tokyo, Japan) and 2.5% FBS, at 33°C.

### Animal models

C57BL/6J mice of six to eight weeks were purchased from Tokyo Laboratory Animals Science (Tokyo, Japan). For the subcutaneous tumor model, mice were inoculated with 1 × 10^5 ^melanoma cells suspended in 100 μl 50% Matrigel (BD Biosciences, Bedford, MA)/phosphate buffered saline (PBS) on the back. For the lung metastasis model, 1 × 10^5 ^melanoma cells suspended in 100 μl saline were injected in the tail vein. All studies involving mice were approved by the institute's Animal Study Committee.

### Vaccination with fixed cells

**Figure 1:**
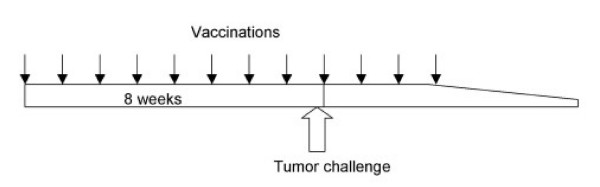
**Experimental plan for vaccination and tumor challenge**. Vaccination was performed once a week for 12 times. Tumor was challenged prior to ninth vaccination on the same day.

Cultured cells in a sub-confluence condition were harvested and fixed in 0.025% glutaraldehyde/PBS for 20 min at room temperature (RT), followed by washing with PBS for three times as described in a previous paper [23]. Vaccination was performed by inoculating 5 × 10^6 ^cells subcutaneously once a week for 8 times before tumor challenge and additional 4 times afterward (Fig. 1). For control, PBS was injected subcutaneously.

### Computed Tomography Scanning

Mice were anesthetized by intrapenetorial injection of 1 mg ketamine hydrochloride and 6.7 μg medetomidine hydrochloride per mouse and subjected to computed tomography scanning using LaTheta LCT-100A in-vivo CT scanner for small animals (Aloka, Tokyo, Japan). For the subcutaneous tumor model, cross-sectional CT scans were taken at 1 mm intervals. Tumor volume was calculated by integration of tumor area in each slice through the tumor by the attached software. For the lung metastasis model, cross-sectional CT scans were taken at 0.5 mm intervals for the whole lung.

### Hybridoma preparation

Fusion of spleen cells harvested from a sacrificed mouse and myeloma cells was performed using Polyethylene Glycol 1500 (Roche, Penzberg, Germany) based on the manufacturer's instruction. Cells were cultured in S-Clone medium (Sanko Junyaku, Tokyo, Japan) supplemented with HAT-media supplement (Sigma-Aldrich Japan, Tokyo, Japan). Selected cell colonies were isolated and conditioned media were harvested and stored at -20°C until use.

### Immunofluorescence of cultured cells

Cultured Tpit/E and B16/F10 cells were fixed with methanol, treated with 0.2% TritonX-100/PBS, washed with PBS, treated with 1% bovine serum albumin (BSA)/PBS, washed with PBS, and treated with the hybridoma-conditioned medium for 30 min at RT. After washing with 0.1% TritonX-100/PBS, 10 μg/mL fluorescein conjugated goat anti-mouse IgG (Chemicon International, MA) diluted in 1% BSA, 0.1% TritonX-100/PBS was applied. After washing with 0.1% TritonX-100/PBS for 3 times, cells were observed by fluorescence and phase contrast microscope. For positive media, immunostaining was repeated after blocking with 100 × diluted normal mouse serum in PBS for 30 min at RT to rule out the possibility of non-specific stickiness to endothelial cell surface molecules including IgG Fc receptors [26].

### Statistical analysis

Correlation between two factors, difference between two groups and difference between survivals of two groups were evaluated by the chi-square analysis, the t-test and the Kaplan-Meier analysis respectively. P values less than 0.05 were considered statistically significant.

## Results

### Inhibition of subcutaneous tumor growth by the Tpit/E vaccination

**Figure 2:**
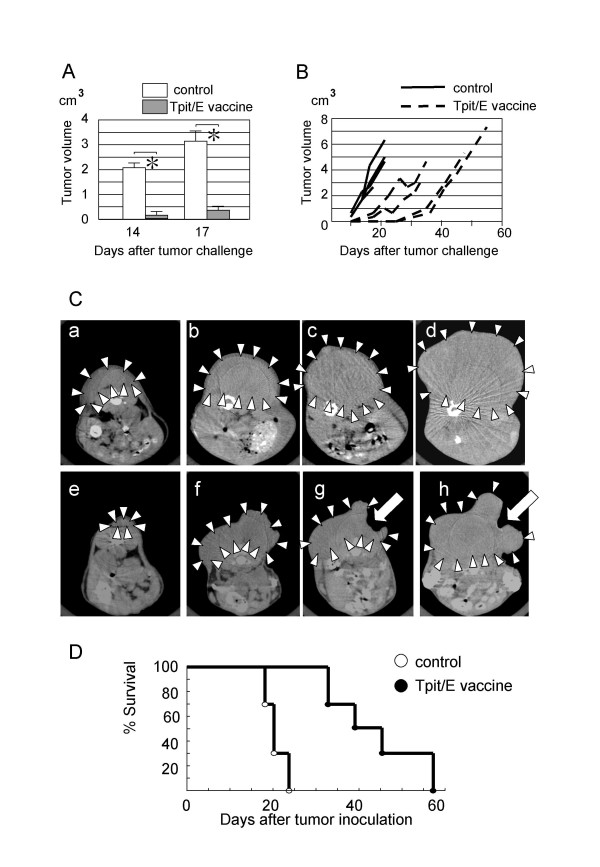
**Tumor growth and survival rate in the subcutaneous tumor model**. A. Subcutaneous tumor volume on the back at day 14 and 17 post tumor challenge. *: p < 0.01 (n = 4). Tumor volume was calculated by integration of consecutive cross-sections obtained by CT scans. B. Time course of subcutaneous tumor volume of each mouse from tumor challenge until death. Tumor growth was apparently inhibited by Tpit/E vaccination. C. Series of CT scan images showing growth of the subcutaneous tumor. (a-d): a control mouse; day 10 (a), 14 (b), 17 (c) and 21 (d) after tumor challenge, (e-h): a Tpit/E vaccinated mouse; day 10 (e), 21 (f), 24 (g) and 28 (h) after tumor challenge. Tumors were indicated by arrowheads. Arrows in panel g. and h. point to necrotic region in the tumor. D. Survival rate after tumor challenge; p < 0.05: Tpit/E vaccine vs. control.

B16/F10 cells were inoculated subcutaneously on the back prior to ninth Tpit/E cell vaccination on the same day and tumor growth was followed by CT scanning twice a week. Experiments ware performed twice and one representative experiment was shown. As shown in Fig. 2A, tumor growth was significantly inhibited in the Tpit/E cell vaccination group compared to control at day 14 and 17 after tumor challenge. Fig. 2B shows a time course of tumor growth in each mouse. Decrease in tumor volume due to massive necrosis in the course was observed in two mice vaccinated with Tpit/E cells. Series of CT images in time course of representative mice from each group are shown in Fig. 2C. Subcutaneous tumor growth of control mice was rapid, while tumors of the Tpit/E vaccinated mice grew slowly with occasional tumor necrosis. Survival period of the Tpit/E vaccination group was significantly longer than control by Kaplan-Meier analysis (Fig. 2D).

### Inhibition of lung metastasis by the Tpit/E vaccination

**Figure 3:**
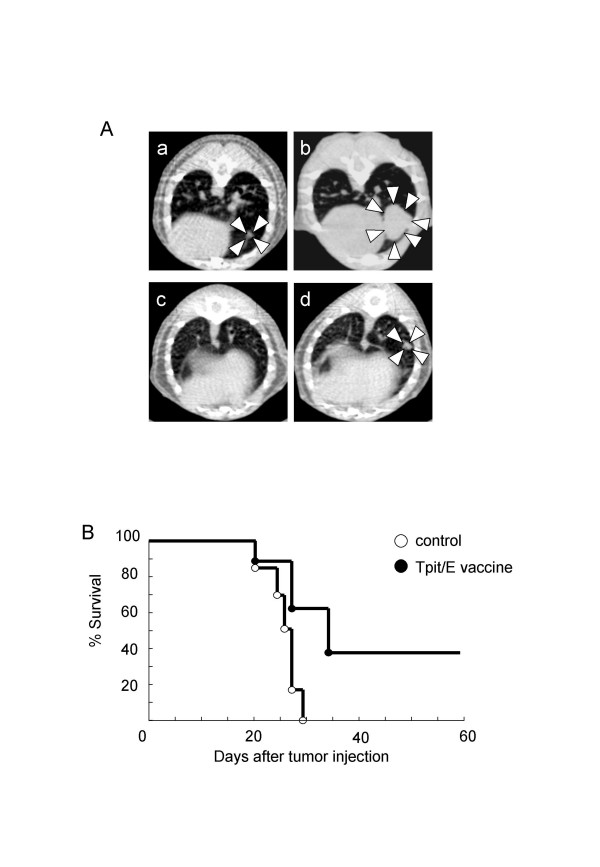
**Tumor growth and survival rate in the lung metastasis model**. A. Series of CT scan images showing development of the lung metastases, (a, b): a control mouse, (c, d): a Tpit/E vaccinated mouse; day 14 (a, c) and 21 (b, d) after tumor challenge. Tumors were indicated by arrowheads. No lung metastasis was observed in panel c. B. Survival rate after tumor challenge, p < 0.05: Tpit/E vaccine vs. control.

B16/F10 cells were injected into the tail vein on the same schedule as the subcutaneous tumor model and development of lung metastasis was followed by CT scanning. At day 7 after tumor challenge, no metastasis was detected in all mice. At day 14, metastases appeared in all of control mice and three out of eight Tpit/E vaccination mice (Table 1). Metastasis appearance rate at this time point was significantly inhibited by Tpit/E cell vaccination. Series of CT images at day 14 and 21 post tumor challenge of representative mice of each group are shown in Fig. 3A. Survival period of the Tpit/E cell vaccination group was significantly longer than control (Fig. 3B).

**Table 1:** Animals with lung metastases at day 14 post tumor challenge

	mice with metastasis	mice without metastasis
control	6	0

T-pit/E vaccine	3	5

Animals with lung metastases at day 14 post tumor challenge; p < 0.05: control vs. T-pit/E vaccine by the chi-square analysis.

### Anti-endothelial cell specific antibody generated in a Tpit/E vaccinated mouse

**Figure 4:**
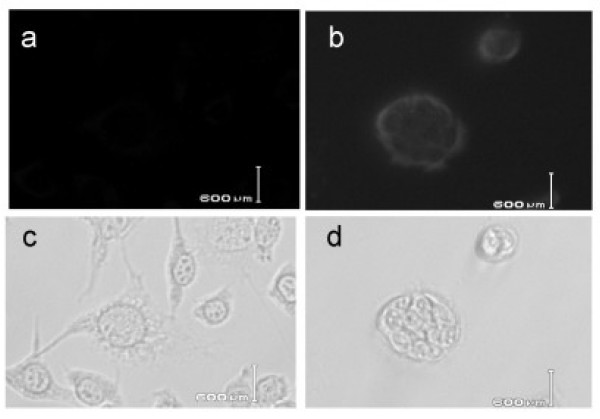
**Antibody to Tpit/E cells generated in the vaccinated mouse**. Immunofluorescence (a, b) and corresponding phase contrast microscopic images (c, d) of cultured cells stained with a representative hybridoma-conditioned media, (a, c): B16/F10 cells, (b, d): Tpit/E cells. Hybridomas were produced from spleen cells of a Tpit/E-vaccinated mouse. The rim of Tpit/E cells was strongly stained, while B16/F10 was not, suggesting that the hybridoma secreted specific IgG reactive to the surface of Tpit/E cells.

To make sure that specific antibodies to Tpit/E cells are generated in a mouse vaccinated with Tpit/E cells, we aimed to obtain Tpit/E specific antibody-secreting hybridoma clones. A surviving mouse in the Tpit/E cell vaccination group of the subcutaneous tumor model was sacrificed at day 45 after tumor challenge. Hybridomas of the spleen cells and SP-2 cells were prepared and the conditioned media were subjected to immunostaining to check the presence of IgG reactive to Tpit/E or B16/F10 cells. Some hybridoma clones were shown to secret antibodies reactive to Tpit/E but not to B16/F10 cells. Images of immunostaining with the medium of a representative clone along with phase contrast images are shown (Fig. 4). Edge of the colonies of Tpit/E cells was strongly stained, while B16/F10 cells were not stained.

## Discussion

So far, vaccination with endothelial cells has been shown to be effective in several mice models using xenogeneic endothelial cells [22,24] and syngeneic endothelial cells [23]. When considering therapeutic application to human, autologous cells may be preferable to avoid host reactions to allogeneic or xenogeneic components in the vaccine. In addition, autologous cell vaccine may have less possibility to induce pathologic autoimmunity due to the tolerance mechanism. However, preparation of a considerable amount of tumor vascular endothelial cells from an individual patient is almost impossible. Therefore, we tested the efficacy of a syngeneic endothelial cell line as a substitute. In addition, cell lines are expected to share characteristics with tumor vascular endothelial cells [25]. These advantages may have contributed to anti-tumor effects on melanoma with high malignancy.

As for cancer models in the previous reports, employed were fibrosarcoma, hepatoma, mammary carcinoma [22], lung carcinoma [22,24], myeloma [24], and colon carcinoma [23]. It is noteworthy that our study showed for the first time the anti-tumor effect of an endothelial cell vaccine against B16/F10 melanoma in both of the subcutaneous tumor and the lung metastasis models. In the course of the subcutaneous tumor growth, occasional tumor necrosis in the Tpit/E vaccinated mice was observed, suggesting occurrence of vascular damage, though further studies are required. Once B16/F10 tumor was challenged, the tumor grew so rapidly and life span was within several weeks without therapy in the present study. Considering a time length required for immune response after vaccination, the anti-tumor effect seemed difficult to detect in a therapeutic setting such as vaccination after tumor challenge. However, anti-tumor effect in a therapeutic setting may be observed if challenged melanoma cells are reduced.

In this study, we aimed to prove that specific antibodies to Tpit/E cells were generated in the vaccinated mice. We thought that positive immunostaining of Tpit/E cells with sera of vaccinated mice may be insufficient because such sera may contain a variety of antibodies including ones against inoculated B16/F10 cells. Therefore, we isolated antibodies by making hybridomas and obtained some clones secreting antibodies reactive with Tpit/E but not with B16/F10 cells. It is obvious that tumor endothelium expresses specific molecules which are not expressed on normal vascular endothelium [27]. Vaccination with Tpit/E cells may have targeted such antigens because vaccination with autologous molecules might not provoke effective immune reaction due to the tolerance. Xenogeneic cells or molecules have been widely employed as vaccines because they tend to break the tolerance [6,10,12,14,17,18,22,24,28]. To show that tumor endothelium specific molecules are targeted, we plan to identify antigen molecules recognized by isolated antibodies in the present study by a proteomics strategy [1]. In addition, effects of the isolated antibodies on tumor vasculature including antibody-dependent cytotoxic activity and also the role of cellular immunity in the response to vaccination should be explored as CTL activities to vaccinated endothelial cells in other settings have been shown [23,24].

To apply the vaccine therapy using an autologous endothelial cell line to human, development of the cell line from a patient is a next subject. Practically, culture of umbilical vein endothelial cells (HUVECs) on a close genetic background may be a good candidate. Recently, a pilot study of HUVECs vaccine in cancer patients with promising results in brain tumors but not in colorectal cancer was reported [29]. No prominent adverse effect observed in the study may facilitate wider application of HUVECs vaccine to various cancers including melanoma. However, some tumor endothelium specific antigens are reported to appear in the vasculature of wound healing tissue [27], possible adverse effects of endothelial cell vaccine on wound healing remains to be clarified.

## Conclusion

Vaccination with a syngeneic endothelial cell line Tpit/E inhibited subcutaneous tumor growth as well as appearance of lung metastasis and elongated survival period of C57BL mice challenged with B16/F10 melanoma, and elicitation of specific antibodies to Tpit/E cells was demonstrated.

## Authors' contributions

KY carried out cell culture and animal experiments.

TN and TN participated in animal experiments.

NY and NY participated in animal experiments and helped to draft the manuscript.
